# Representation of individual elements of a complex call sequence in primary auditory cortex

**DOI:** 10.3389/fnsys.2013.00072

**Published:** 2013-10-30

**Authors:** Mark N. Wallace, Jasmine M. S. Grimsley, Lucy A. Anderson, Alan R. Palmer

**Affiliations:** ^1^MRC Institute of Hearing Research, University Park, Nottingham, UK; ^2^Department of Anatomy and Neurobiology, Northeast Ohio Medical University, Rootstown, OH, USA; ^3^Ear Institute, University College London, London, UK

**Keywords:** vocalization, cortical column, communication, medial geniculate body, guinea pig, temporal processing

## Abstract

Conspecific communication calls can be rhythmic or contain extended, discontinuous series of either constant or frequency modulated harmonic tones and noise bursts separated by brief periods of silence. In the guinea pig, rhythmic calls can produce isomorphic responses within the primary auditory cortex (AI) where single units respond to every call element. Other calls such as the chutter comprise a series of short irregular syllables that vary in their spectral content and are more like human speech. These calls can also evoke isomorphic responses, but may only do so in fields in the auditory belt and not in AI. Here we present evidence that cells in AI treat the individual elements within a syllable as separate auditory objects and respond selectively to one or a subset of them. We used a single chutter exemplar to compare single/multi-unit responses in the low-frequency portion of AI—AI(LF) and the low-frequency part of the thalamic medial geniculate body—MGB(LF) in urethane anaesthetized guinea pigs. Both thalamic and cortical cells responded with brief increases in firing rate to one, or more, of the 8 main elements present in the chutter call. Almost none of the units responded to all 8 elements. While there were many different combinations of responses to between one and five of the elements, MBG(LF) and AI(LF) neurons exhibited the same specific types of response combinations. Nearby units in the upper layers of the cortex tended to respond to similar combinations of elements while the deep layers were less responsive. Thus, the responses from a number of AI units would need to be combined in order to represent the entire chutter call. Our results don't rule out the possibility of constructive convergence but there was no evidence that a convergence of inputs within AI led to a complete representation of all eight elements.

## Introduction

Many individual animal communication calls carry contextual information, such as warnings (Slobodchikoff et al., 1991; Greene and Meagher, 1998; Gadziola et al., 2012). These types of calls provide useful tools for investigating how meaning, or discrimination among these sounds, is represented in the brain (Kanwal et al., 1994; Pfalzer and Kusch, 2003; Wang et al., 2007). This simplistic single call system is also used by humans when people scream, or shout single words such as “stop” or “help.” However, human speech is typically made up of temporally separate acoustic components; these can either be individual words or strings of syllables within a word (Zipf, 1935). Individual syllables are often composed of constant frequency vowels with a consonant at the beginning and/or end but some languages also include clicks (Fulop et al., 2003). Some animals also produce communication calls comprised of separate acoustic elements either alone or combined into syllables (Berryman, 1976; Wohlgemuth et al., 2010; Berwick et al., 2011; Grimsley et al., 2011a). These call types allow us to study how natural sequences of sounds are coded in the brain (Esser et al., 1997; Bolhuis and Gahr, 2006). One example of a call produced as a series of separate syllables is the guinea pig chutter call and this call is typically produced in bouts that can last for over a minute. The chutter is a mildly aversive call that is produced as a result of unwelcome attention or as an indication of mild irritation (Berryman, 1976). Most of its energy is contained in low-frequencies and it is typically produced at relatively low sound levels compared to more strongly emotive calls such as the scream or whistle. Each chutter contains a series of syllables formed from at least one of the three basic elements that form the basis of most animal and human vocalizations: steady-state, harmonically related frequencies, frequency modulations, and noise bursts (Eggermont, 2001). Hence the chutter provides a rich stimulus with which to probe cortical processing of natural sound sequences. Calls containing these types of spectrotemporal components occur in many species and it has been suggested (Suga et al., 1978) that they be analyzed in terms of acoustically distinct elements which are able to generate a specific response by neurons. These elements are analogous to the phonemes of human speech but may not have any meaning associated with them. In this study, we identified distinct acoustic elements that were able to elicit a clear response from the guinea pig auditory cortex (AI). The chutter used here had 12 acoustic elements but only 8 of them could elicit responses at the sound levels routinely used. The particular exemplar used here was chosen because it contained five syllables: one that was a constant frequency element, one that was a frequency modulated element, two that were clicks and one more complex syllable that contained all three elements.

Some neurons in AI respond to communication calls by firing spikes in temporal patterns that mimic the waveform envelopes of the calls. This is particularly true of rhythmic calls such as the frequency-modulated (FM) twitter calls in the marmoset (Wang et al., 1995) the FM chirrup and whistle string or the amplitude-modulated, constant-frequency purr of the guinea pig (Wallace et al., 2005; Grimsley et al., 2012). It is also true of broadband click trains in the marmoset (Lu et al., 2001) and tooth chatter in the guinea pig (Grimsley et al., 2011b). Individual neurons in guinea pig AI can provide faithful representations (isomorphic responses) of the waveform envelopes of many repetitive calls, even when calls are of long duration (more than a second). However, that did not seem to be true for the chutter call which has an irregular sequence of syllables. Neurons in AI seemed unable to accurately represent the waveform envelope of either the chutter or the related chut call (Wallace and Palmer, 2009; Grimsley et al., 2012). This is in contrast to one of the rostral belt areas (area S) where units can provide very accurate representations of both the chutter and chut envelopes. We hypothesize that individual AI neurons are unable to accurately represent the envelope of calls comprised of elements that are acoustically different. This is a restricted expression of the more general hypothesis put forward previously (Nelken et al., 2003) that the role of AI is in splitting sound within a frequency channel into separate auditory objects that can then be processed by higher auditory centers. This presupposes that an individual element within a call with a unique spectral and temporal structure can be defined as an auditory object (Griffiths and Warren, 2004). We propose that AI neurons do not normally engage in the integration of a temporal sequence of different elements within complex calls and that this may occur in belt areas such as area S. To test our hypothesis about temporal integration of the elements within communication calls we have measured the cortical responses to an exemplar of the chutter among cells optimally matched to respond to its spectral energy (CFs ≤ 1.5 kHz). We have also compared frequency-matched responses in the thalamus in order to make inferences about the types of processing occurring in the cortex.

## Materials and methods

### Surgical procedures

Twenty nine pigmented guinea pigs of both sexes and weighing 430–900 g were bred in-house. Anesthesia was induced with urethane (1.1 g/kg in 20% solution, i.p.) supplemented as necessary by 0.2 ml doses (i.m.) of Hypnorm (VetaPharma Ltd., Leeds, UK; fentanyl citrate 0.315 mg/ml; fluanisone 10 mg/ml) to maintain abolition of the forepaw withdrawal reflex. Cannulae were inserted into the auditory bulla to equalize the pressure in the middle ear. After making a craniotomy and removing the dura, electrodes were inserted into the low-frequency end of the primary auditory cortex—AI(LF) mainly in a direction that was orthogonal to the surface, but some tangential tracks were also made. Electrodes were inserted into the low-frequency area of the medial geniculate body of the thalamus—MGB(LF) stereotaxically after removing the dura over the overlying cortex. Electrolytic lesions were made in tracks involving thalamic or cortical recordings and sections of the brain were stained to demonstrate the metabolic marker cytochrome oxidase (thalamus) or Nissl substance with Cresyl Violet (cortex) as described previously (Anderson et al., 2007). This allowed us to confirm the location of our recordings within the medial geniculate body or cortex. All experiments were performed under the terms of a project license issued under the United Kingdom Animals (Scientific Procedures) Act 1986 and following approval by the University of Nottingham Ethical Review Committee.

### Stimulation and recording

Auditory stimuli were delivered diotically through sealed acoustic systems, comprising modified Radio Shack 40-1377 tweeters joined via a conical section to a damped, 2.5 mm diameter, probe tube that fitted into the speculum. All stimuli were presented binaurally. The system was calibrated in each experiment by inserting a probe tube microphone close to the tympanic membrane. The search stimuli, generated by an array processor (Tucker-Davis Technologies AP2), were pure tones (duration 100 ms) gated on and off with cosine squared ramps lasting 8 ms and with a repetition period of 800 ms. When a unit was isolated, we determined the minimum response threshold and the characteristic frequency (CF: the frequency giving the lowest threshold) by plotting a frequency response area (FRA). A total of up to 961 tone pips (100 ms duration, 8 ms rise/fall times) were randomly interleaved and presented once at intervals of 600 ms. Attenuations of 0–100 dB in 5 dB steps were used and the frequencies ranged over 6 octaves in 5 steps per octave. The CF was estimated from the previous unit in the track and tones were generated to cover two octaves above and four octaves below this value. Discriminated spikes were counted in a 100 ms window that started 10 ms after the stimulus onset.

A single exemplar of chutter was presented by using the same digitized recording (44.1 kHz) used in a previous study based on the mid to high-frequency end of AI (Wallace and Palmer, 2008). This particular exemplar was not chosen because it was a typical example of chutter, but because it contained four different types of syllable within a period of less than a second. The chutter used had three main bursts of sound (each lasting 100–150 ms) followed by two much quieter sounds in the form of clicks. In our previous study, the chutter was presented at a constant high sound level: when units with low thresholds were recorded very quiet elements of the call also produced a response thus increasing the number of potential response combinations. Within the group of units responsive to the chutter there was a wide range of pure-tone minimum thresholds (from 10 to 63 dB SPL). In the present study, the chutter was presented at a customized sound level to each isolated unit with the attenuators set to a level 20 dB above that used to obtain the pure tone threshold and was repeated 30 times at 3 s. intervals. This reduced the impact of the large range of level sensitivity among cortical units by ensuring that each was stimulated at ~20 dB above their call threshold. This made it easier to compare the temporal aspects of the responses.

Recordings were made with custom made, glass-insulated, tungsten electrodes (Bullock et al., 1988) which generally have tip impedances of 0.5–4 MΩ at 1 kHz. Electrodes were advanced by a piezoelectric motor (Burleigh Inchworm IW-700/710) in steps of 2.5 μm. Whenever possible single units were isolated using a voltage discriminator which only captured the larger spikes, but in some cases we also recorded from a small number of units which had spikes of a similar size and form. Responses were plotted as peristimulus time histograms (PSTHs) based on the 30 repetitions with 5 ms binwidths and a unit was considered to have responded to a particular part of the call if the number of spikes in a bin exceeded two standard deviations of the background and had a minimum of 10 spikes.

## Results

### Tuning for pure tones affects responsiveness to the chutter call

**Figure 1:**
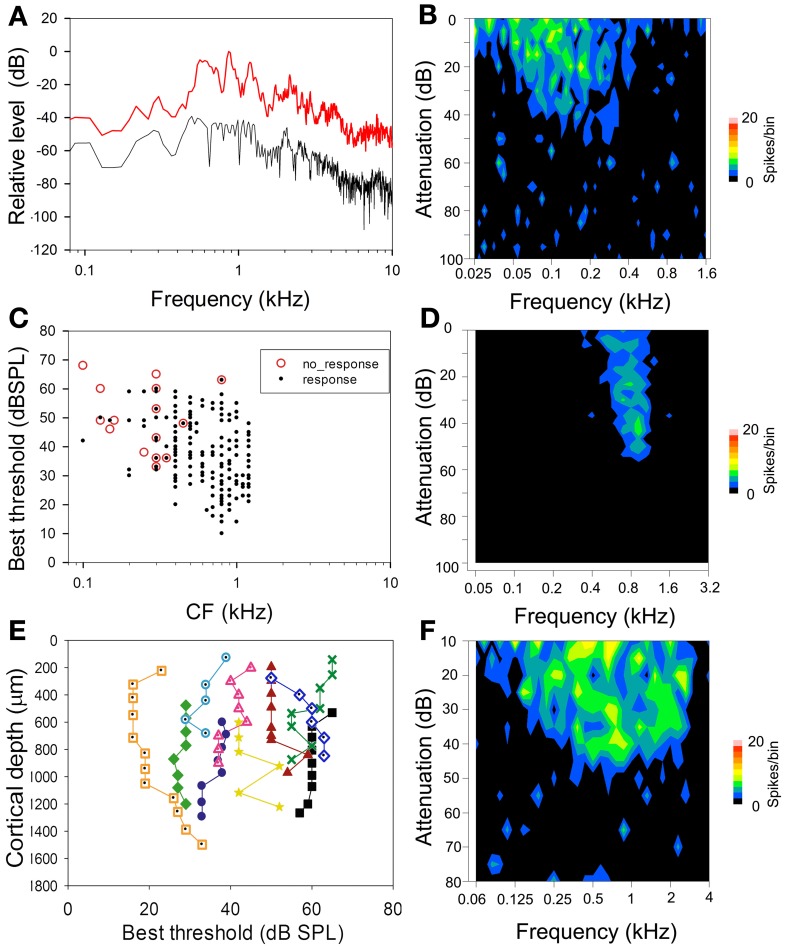
**(A)** High resolution spectral analysis of the chutter based on the mean of 50 ms segments of the three main phrases in the call. The graphs show the maximum (red) and minimum (black) sound levels for each frequency plotted relative to the loudest sound level in the call. Most of the energy is contained within the frequency range 0.5–1.5 kHz and this is the range of CFs for most of the units selected in this study. **(B)** Frequency response area (FRA) of a unit with a CF of 0.13 kHz which did not respond to the chutter call. **(C)** Distribution of CFs and best thresholds of the units recorded in this study. The red circles indicate units which did not respond to the chutter call. There is a broad range of thresholds for units at each CF. The unresponsive units have a CF of below 0.4 kHz or a high threshold. **(D)** Narrowly tuned FRA from a unit with a CF of 0.8 kHz that responded reliably to the chutter call. **(E)** Line plots of changes in the best threshold of units in 10 separate orthogonal tracks. Overall there is a range of about 50 dB in the best thresholds shown, but the thresholds don't vary by more than 20 dB in any one track. **(F)** Broadly tuned FRA from a unit with a CF of 0.7 kHz that showed a strong response to the chutter and illustrates the fact that CF, best threshold and width of tuning may all be factors in determining to what degree a unit responds to the chutter call.

The probability of a unit responding to a communication call was largely predicted by its response to pure tones. Units with FRAs that overlapped with the main spectral range of the chutter call were more likely to respond to the call. Most of the spectral energy within the chutter call was in the range of 0.5–1.5 kHz (Figure 1A). For this reason we selected units for the present study with a CF of ≤1.5 kHz (Figure 1C); a total of 264 units in AI(LF) and 46 units in MGB(LF) met this criterion. Units with CFs of <0.4 kHz in AI(LF) and MGB(LF) were less likely to respond than those with CFs between 0.4 and 1.5 kHz (Figure 1C): of the18 AI(LF) units that did not respond to the chutter call 16 had CFs of between 0.1 and 0.35 kHz. Similarly for the seven units in MGB(LF) that did not respond five had CFs between 0.1 and 0.35 kHz. An example of the FRA for a unit with a CF of 0.13 kHz, which did not respond to chutter, is shown in Figure 1B. This unit was unresponsive to pure tones of above 0.4 kHz. By contrast a more narrowly tuned unit with a CF close to the center of the spectral range within the chutter responded consistently to the chutter (Figure 1D), while a more broadly tuned unit that responds to tones across all of the main spectral range in the chutter (Figure 1F) responded even more strongly to the chutter. Fisher's Exact Test showed a negative association between CF and responsiveness for AI(LF), *P* = 2.10^−10^ when responses from units with CFs less than 0.4 kHz were compared to those with CFs of 0.4–1.5 kHz. AI(LF) units were located within 1.5 mm caudal from the most anterior portion of the pseudosylvian sulcus. Within an orthogonal track adjacent units had a similar CF (always within half an octave) and pure tone threshold (adjacent units had thresholds that were within 10 dB of each other). Some orthogonal tracks had units with very similar thresholds and none showed a range of more than 20 dB (Figure 1E).

### Responses to acoustically distinct elements

**Figure 2:**
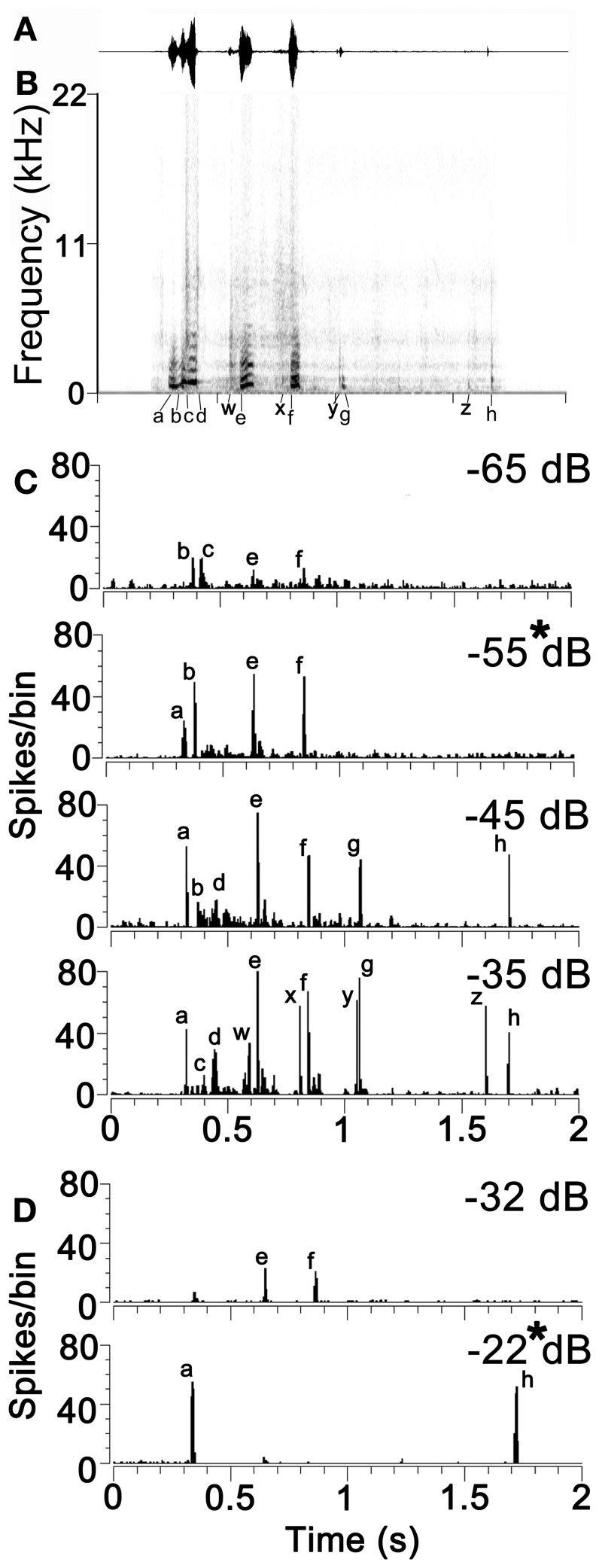
**(A)** The waveform and **(B)** the spectrogram of the example of chutter used in this study. The eight temporal elements of the call that are capable of producing a neuronal response at low sound levels are indicated by the letters (a) to (h) below the time axis. Quieter elements of the call that only produce a neuronal response at relatively high sound levels are indicated by the letters (w) to (z). **(C)** Responses of a unit in AI(LF) to the chutter call presented at four different attenuation levels from −65 dB (quietest) to −35 dB (loudest). Individual peaks in the response corresponding to the different temporal elements of the spectrogram are indicated by the same letters as in panel **(B)**. This unit had a CF of 1 kHz and a pure tone threshold of 25 dB SPL. **(D)** Separate unit in AI(LF) which showed different responses to chutter at two different levels of attenuation. This unit had a CF of 0.4 kHz and a pure tone threshold of 58 dB SPL.

The exemplar of chutter used in this study had three main syllables (each lasting 100–200 ms) followed by a series of much quieter and briefer clicks. Each syllable was separated by at least 100 ms from the others and had one to four elements each of which drove neurons in the cortex at the relatively low sound levels used. The number of elements that produced a response depended on the response characteristics of the unit and the sound level of the stimulus. The structure of the call is illustrated by the waveform and spectrogram in the first two panels of Figure 2. The first syllable was the most complex part of the call. It was composed of the following sequence: (a) a strong pulse of a static low-frequency (fundamental of 0.5 kHz) harmonic complex which merges into (b) a brief frequency modulated ramp that ends in (c) a noisy transition to another constant frequency pulse with four harmonics and a higher fundamental frequency (0.8 kHz) than (a). This ends abruptly with a broad band noise burst (d). There was then a gap of about 100 ms before the second syllable which started with a quiet noise burst (w) followed by a shallow frequency modulated glide (e) similar to (b) but with a longer duration and more power. There was a gap of 150 ms before the third syllable which again started with a quiet noise burst (x) and was followed by a constant frequency burst similar to (a). There was then another 150 ms gap before a double click (y) (g) where only the second click had enough power to activate neurons in the forebrain at low sound levels. There was then a gap of about 500 ms before another quiet click (z) and then a louder click (h). Altogether 8 elements (a–h) were identified that could elicit a specific cortical response (spike rate at >2 s.d. above background) at the sound levels used in this study. There were also four quiet elements which only activated neurons at higher sound levels than were routinely used in this study (w–z).

Increasing the sound level generally increased the number of elements which elicited a response from thalamic and cortical cells. This was specifically tested in 21 units where the call was presented at 20 dB above pure tone threshold and then at 40 dB above threshold. For every unit the higher sound level produced a response to a higher number of elements. The mean number of elements responded to at the lower sound level was 4 while at the higher sound level it was 8.5. This was a highly significant difference (paired *t*-test *P* = 2.3 × 10^−9^). The effect of changing sound level is illustrated in Figure 2C which shows the response of a unit in AI with a CF of 0.8 kHz and a threshold of 22 dB SPL. When the chutter was presented at increasing sound levels by decreasing the attenuation from −65 to –35 dB there was an increase in the number of elements producing a response from 4 to 11. This was not a simple linear relationship because both the elements b and c produced a response from the unit at the lowest sound level, but only one or the other produced a response at the higher levels. Another example is shown in Figure 2D for a high threshold unit with a CF of 0.4 kHz which had very different responses at two sound levels only 10 dB apart. In order to minimize these effects of sound level on the cortical responses we always presented the chutter at a sound level corresponding to 20 dB above the pure tone threshold (panels marked by asterisks in Figure 2). In practice this meant that the peak sound level of the chutter presented during the recordings ranged from 30 to 83 dB SPL with the median of 57 dB SPL.

**Figure 3:**
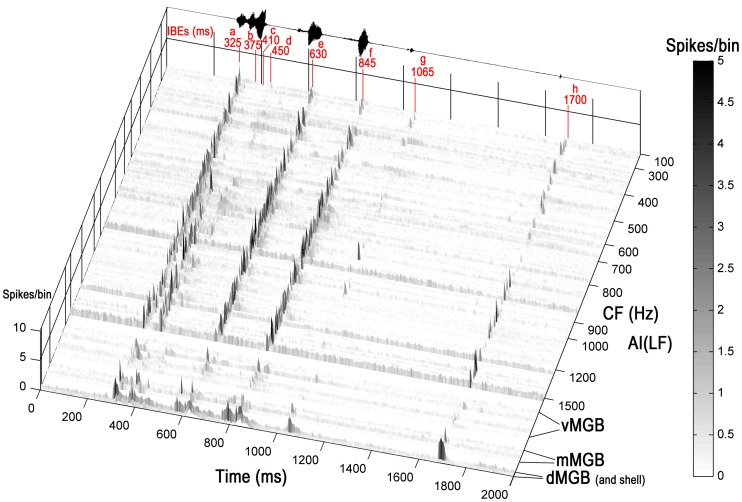
**Three-dimensional heat plot of the firing rate for all the units in the cortex and thalamus arranged in tonotopic order.** Units from three divisions of the MGB are shown at the base of the figure and each division is separated by a white line. At the top of the plot there is a representation of the call waveform and the position of the eight acoustically distinct elements is indicated along with their mean latency relative to the start of the stimulus file given in ms. Most units respond by giving brief increases in their firing rate at times that correspond to one or more of the elements in the call. The firing rate is given as spikes/bin/stimulus repeat.

**Figure 4:**
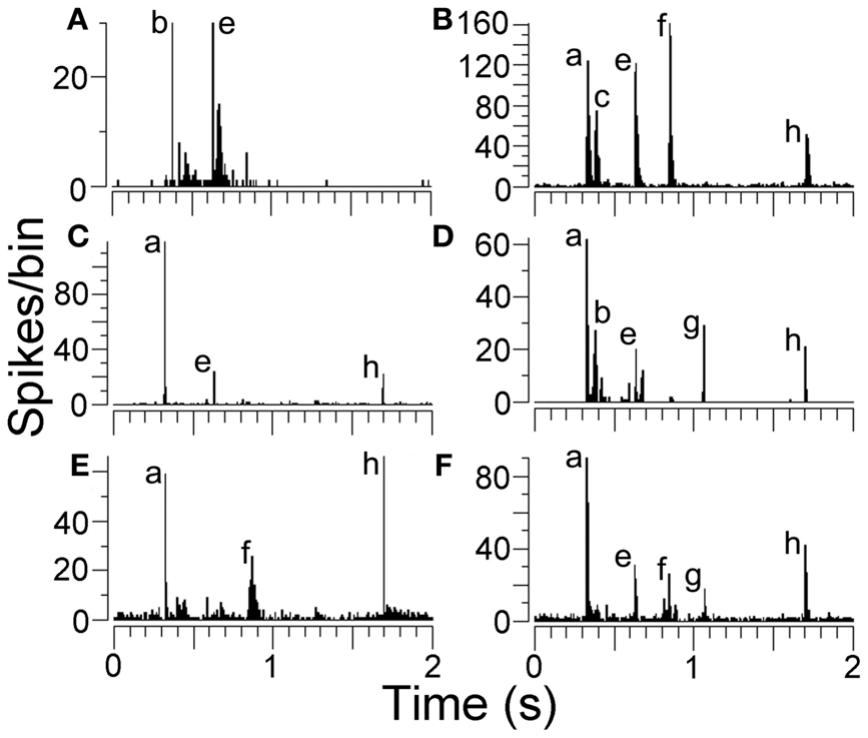
**(A–F)** Peristimulus time histograms (PSTHs) showing examples of responses from AI(LF). Some units only responded to two or three elements while others responded to five elements as indicated by the labels.

Units in AI(LF) that responded to the chutter call responded with sharp and relatively brief increases in their firing rate to one or more of the 8 elements. The element could be identified by the latency of the response. This is illustrated in Figure 3 which shows a three-dimensional plot of the firing rate for all the units in the cortex and thalamus arranged in order of increasing CF. At the top of the plot there is a representation of the call waveform and the position of the eight elements is indicated along with the mean latency of the cortical responses to the individual elements relative to the start of the stimulus file. The 246 units in AI(LF) responded to between 1 and 8 elements by showing a rapid but usually brief (≤30 ms) increase in firing rate (e.g. Figures 4C,E,F). Occasionally a more sustained response to an individual element occurred (<50 ms) as shown for the (e) element in the individual PSTHs in Figures 4A,D. Offset responses were rare and, when they did occur, were relatively weak. Cortical units respond to pure tones at CF with a relatively small range of latencies [about 10–20 ms; (Wallace et al., 2000)] and this was also to some extent true of the responses to the acoustic elements. Thus, the mean onset latencies for the cortical population were: (a), 325 ms; (b), 375 ms; (c), 410 ms; (d), 450 ms; (e), 630 ms; (f), 845 ms; (g), 1065 ms; (h), 1700 ms. The thalamic onset responses were typically 5 to 15 ms earlier and were: (a), 310 ms; (b), 360 ms; (c), 405 ms; (d), 445 ms; (e), 615 ms; (f), 835 ms; (g), 1040 ms; (h), 1685 ms. Individual units could vary from this by up to ±10 ms.

**Figure 5:**
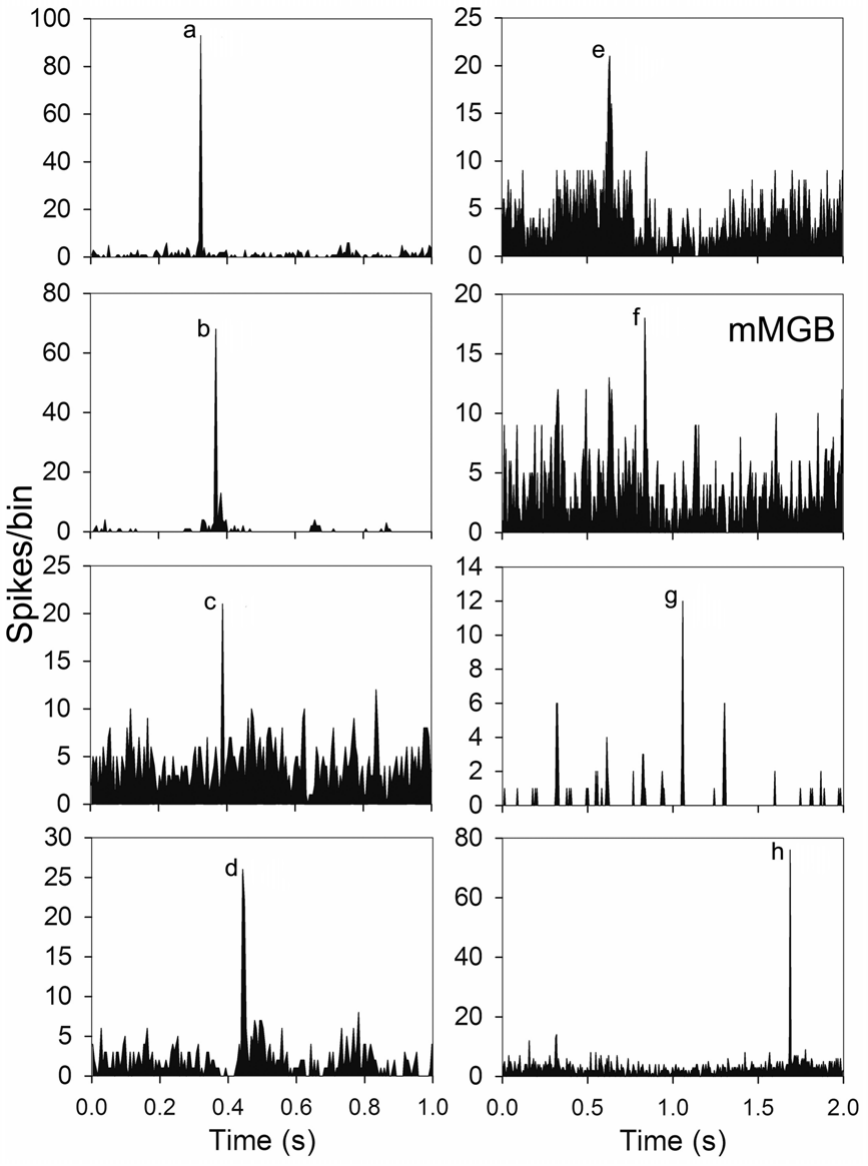
**Examples of PSTHs from seven units in AI(LF) and one from the medial MGB (mMGB) each of which showed a response to a different single element**.

Some units were highly selective and only responded to one element in the call. Units in AI(LF) responded selectively to 7 out of the 8 individual elements: (a,b,c,d,e,g,h) and examples of these units are shown in Figure 5. Out of the 264 units in AI(LF) 29 (11%) responded only to a single element although the majority of these were to element (a) (17/264; 6.4%). The other 6 elements had selective responses from 1 to 3 units each. The only element which did not produce a selective response in any cortical units was element (f), but one of the units in the medial MGB gave a small selective response to element (f) (see Figure 5) so it is likely that there may also have been a small number of (f)-selective cortical units which we did not sample. The lack of a clear selective response to (f) in the cortex may have been due to the close spectral similarity between elements (a) and (f): any unit that would respond to (f) would already have responded to (a). The reverse does not apply because many cortical units tend to show an onset response when they are not optimally stimulated and so will respond to the first presentation of a brief stimulus, but not to subsequent presentations within the same series (Wang et al., 2005). The finding that there were 29 units that only responded to one element and only one unit that responded to all eight elements emphasises the propensity of AI(LF) to respond to individual elements rather than all the elements within the call.

Spectral sensitivity also had a role in determining which units would respond to some of the elements. This was true for elements b and c both of which produced very few responses among units with CFs of <0.7 kHz. Thus, of the units with a CF <0.7 kHz that were presented with the chutter stimulus only 4% (5/124) responded to elements b or c while for units with CFs ≥0.7 kHz ≤1.5 kHz 65% (91/139) of units responded to elements b and/or c. This is illustrated in Figure 3. For the other six elements the CF of the unit did not appear to have any clear correlation with the presence of a response.

One particularly interesting aspect of the responses to the first four elements was that they could be just as temporally precise and contain as many spikes as the responses to the last four elements despite the fact that the first four elements were all fused together in one syllable whereas the last four elements were all in separate, temporally isolated syllables or clicks (Figure 3). A few examples of this are shown in more detail in Figures 4B,D where the responses to elements (b) and (c) are almost as large as those to the later elements despite coming less than 90 ms after the response to (a). In addition, even when the response to element (a) is suppressed, the response to element (b) can be almost as large as to any subsequent elements (Figure 4A). When the mean size of the response to the (b) element was compared after a response to element (a) (21 spikes/bin) with the mean size after no response to element (a) (19 spikes/bin) there was no significant difference (Student's *t*-test *P* = 0.74). This implies that the effects of adaptation may not be very effective in modulating the response to the early elements. A total of 216 units responded to element (a) and of these 47% (102/216) also responded to at least one other element in the first syllable.

### Similarity of responses to chutter in the MGB(LF) and AI(LF)

**Figure 6:**
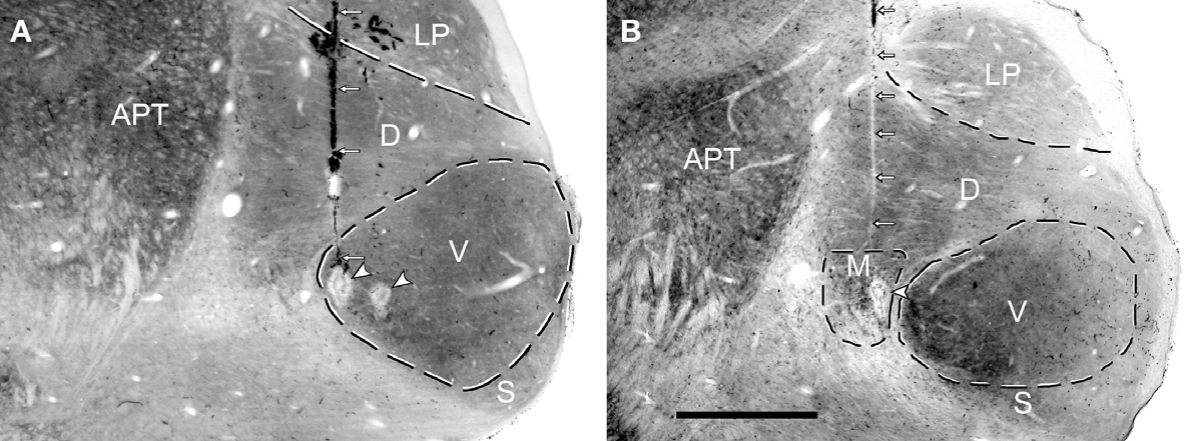
**Coronal sections through the medial geniculate body (MGB) that have been stained for cytochrome oxidase activity.** High enzyme activity occurs in the anterior pretectal nucleus (APT) and in the ventral (V) division of the MGB while relatively low levels occur in the lateroposterior (LP) nucleus and shell division of the MGB. The dorsal division (D) of the MGB has intermediate enzyme activity while the medial division (M) of the MGB has a striated appearance because of bundles of fibers. **(A)** A single electrode track (small white arrows) filled with darkly stained red blood corpuscles terminates in an electrolytic lesion (white arrowhead) at the medial edge of the ventral MGB. The edge of another electrolytic lesion is slightly more lateral but the track associated with this lesion cannot be seen in this section. The CF of the unit recorded at the medial lesion was 0.55 kHz while the CF of the more lateral lesion was 1.4 kHz. **(B)** The pale line of damaged tissue indicating an electrode track (white arrows) ends in an electrolytic lesion (arrowhead) in the medial division. The CF of the unit at the lesion was 1.5 kHz.

**Figure 7:**
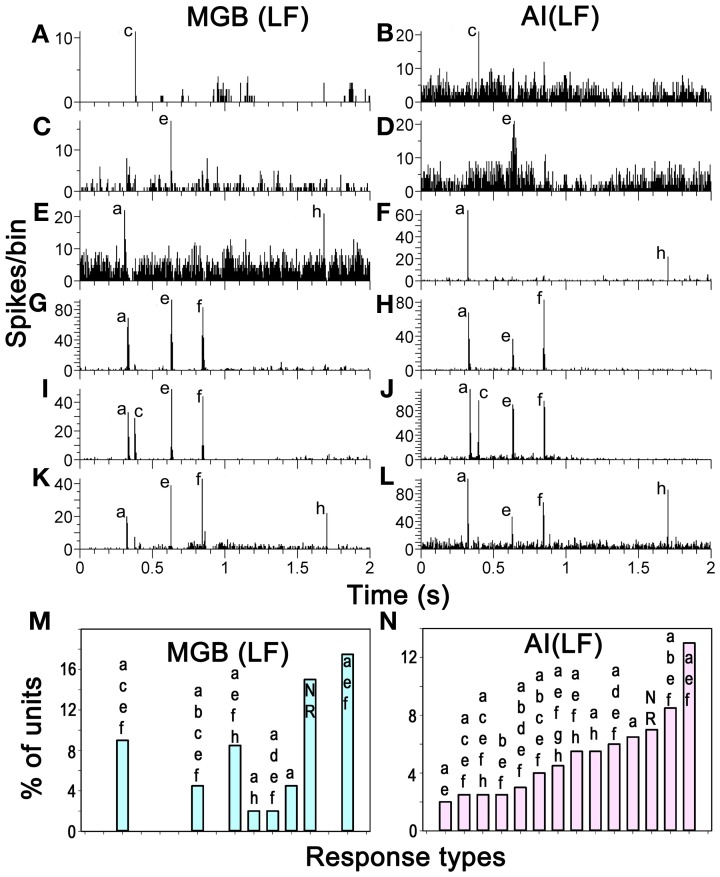
**(A–L)** PSTHs showing matched pairs of distinct response combinations to the chutter by units in MGB(LF) and AI(LF). Some units only responded to one element while others responded to two, three or four elements as indicated by the labels. **(M, N)** Proportions that particular response combinations made in each structure. The 14 most common combinations in AI(LF) are shown as the proportion of the total number of units and the corresponding proportion for each combination is shown for MGB(LF).

The positions of the chutter sensitive units in the MGB(LF) were located by the placement of lesions and histological reconstruction of the electrode tracks as illustrated in Figure 6. This permitted the identification of 4 main MGB divisions based upon their distinctive staining for cytochrome oxidase. Over half of the units (30/46) were in the ventral division, but some were in the medial division (11) and others were in the dorsal division (3) or the shell (2). Many types of response combination were identified in the MGB(LF) and 6 examples are shown in the left half of Figure 7. Many of the same response combinations were found in AI(LF) and corresponding examples from the cortex are shown on the right side of Figure 7. The most common type of response in MGB(LF) (8/46 units; 17%) was a response to the onset of the three main bursts of sound (elements a,e,f) and an example is shown in Figure 7G. This was also the most common response type in AI(LF) (34/264 units; 13%) and an example is shown in Figure 7H. Units in MGB(LF) responded selectively to individual elements in the same way as the cortex. Thus, in Figure 7A the thalamic unit responded selectively to element (c) while the unit in Figure 7C responded selectively to element (e). In the thalamus six of the units (13%) responded to only 1 element and this was similar to the proportion in the cortex (11%). Selectively responding to one element alone was a feature of both thalamic and cortical units and seemed equally likely in either structure. Other thalamic units responded to different combinations of elements (Figures 7E,I,K). These combinations also occurred in the AI(LF) (See Figures 7F,J,L). The mean number of elements that a unit responded to in the MGB(LF) was 3.7 while in the AI(LF) it was 3.5. These means were not significantly different (Student's *t*-test, *P* = 0.32).

A total of 47 different element response combinations were recorded in AI(LF) while in MGB(LF) there were 19 separate combinations. Among the 19 combinations in the thalamus eight were not found among the 246 responses recorded in AI(LF). This high degree of variability for MGB(LF) implies that if more units had been recorded there would have been a similar number of combinations as that seen in the cortex. To test this we counted the number of unique response combinations among 46 randomly chosen AI(LF) units and found 22. This was similar to the number in the MGB(LF) and implied that there is a similar diversity of responses in both structures. The differences between the two populations may just be due to sampling. The combination types were not uniformly distributed as some types of combination response were more common than others. In AI(LF) we recorded more than 4 (2%) examples of responses to each of 14 combinations and collectively these 14 types formed 72% of the cortical units. Of these 14 types, there were 8 (57%) where at least one example was also recorded in MGB(LF). The proportion that each of these 14 types made to the total number of units in each structure is shown in the last two panels of Figure 7. The distributions show a similar trend and the differences may only represent inadequate sample size in (MGB(LF).

**Figure 8:**
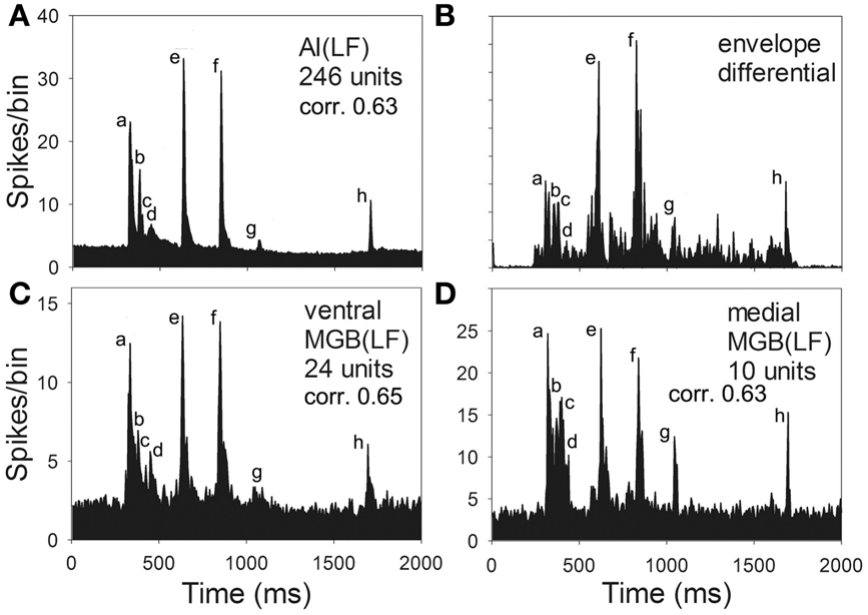
**Mean response to chutter derived from the population of low-frequency units (CF <1.5 kHz) recorded in (A) AI(LF) (C) ventral and (D) medial MGB.** The mean responses all look similar to each other and to the first differential of the waveform envelope shown in panel **(B)**. The correlation values between each of the population responses and the first differential are also very similar.

To compare the population responses to the chutter in MGB(LF) and AI(LF) a mean response was plotted for AI(LF) and for the medial and ventral divisions of MGB(LF). These are shown in Figure 8 along with a representation of the first differential of the half-wave rectified chutter envelope. We had previously used the first differential of the waveform as a template to measure the strength of correlation between exemplars of each of the 10 guinea pig calls and the cortical responses. Isomorphic responses had high correlation values when the first differential of the chutter waveform was compared with the unit response (Grimsley et al., 2012). In AI(LF) the mean response is very similar to the response in ventral and medial MGB(LF) and all are similar to the differential of the waveform envelope except that the response to element (c) is more obscured by the background activity in AI(LF). Thus, there was no evidence to indicate that there was any systematic difference in the representation of chutter in frequency-matched areas of AI and MGB.

### Modular responses to chutter in AI(LF)

The 47 response types to chutter in AI(LF) were not randomly topographically organized. Instead, units recorded over a distance of up to 1 mm from a single track oriented either orthogonally or tangentially to the cortical surface often exhibited similar responses to the chutter providing they were in the upper layers (I–IV). Out of 29 orthogonal tracks where 4 or more units were recorded, 15 showed similar responses throughout the upper part of the track from the first unit, at a depth of 100–200 μm, down to about 1100 μm which corresponds to the base of layer IV (Wallace and Palmer, 2009). Three of the orthogonal tracks contained units which did not respond to the chutter (but which did respond to tones), while 11 tracks contained units some of which were different from each other in a way that suggested separate thalamic inputs. Only one track showed similar responses from layer II into the depths of layer V, but this was mainly because we recorded very few units that were deeper than the upper edge of layer V.

**Figure 9:**
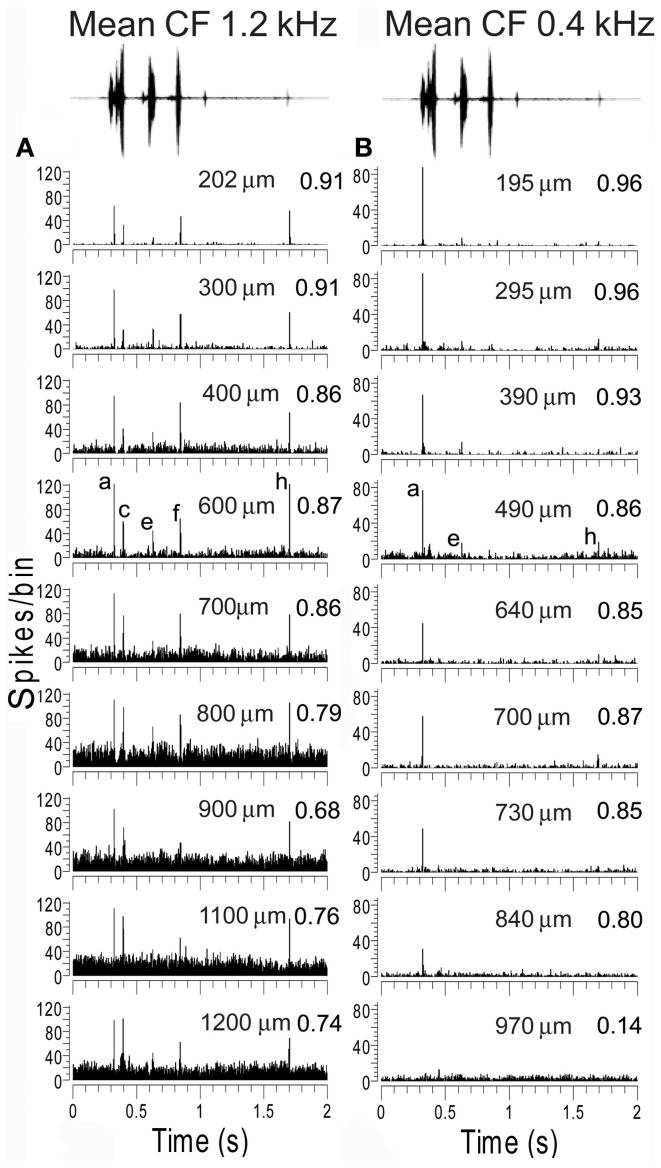
**Recordings from units at various depths from two experiments where an electrode was inserted into AI(LF) in tracks oriented orthogonally to the surface.** The mean CF of units in each track is shown at the top as well as the waveform of the chutter. The elements responded to by each unit within one track are remarkably similar. The units in track **(A)** all respond to the same five elements although the response to element (e) is very small in a few of the deepest units. Their similarity is indicated by the value on the right hand side of each panel which shows the correlation between the PSTH and the mean PSTH for that track. The units in track **(B)** primarily give an onset response to element (a) although the response gets smaller with increasing depth until by the deepest unit there is no longer any significant response to this element. The upper units also show a response to element (e) and two of the units also give a small response to (h). Thus, although not identical the responses are similar as shown by the correlation values at the right of each panel.

Examples of tracks with similar responses are shown in Figure 9 which shows consecutively recorded PSTHs for two orthogonal tracks in AI(LF). Each plot shows the responses from units recorded at 9 or 10 different depths in sequence. In track A the units all gave a strong response to elements (a), (c), (f), and (h) and most gave a small response to element (e). To quantify their similarity each PSTH was correlated with the mean PSTH for the track. All the correlation values were 0.68 or above as shown by the numbers at the right hand side of each panel. By contrast, in track B all of the first eight units gave their largest response to element (a) and a few gave a small response to elements (e) or (h). Again the correlation values for each PSTH in relation to the track mean are given and all are 0.8 or above except for the last unit which barely responded to the call. Tracks such as these two indicate that there are some functionally defined modules in AI(LF), at least in the upper layers.

**Figure 10:**
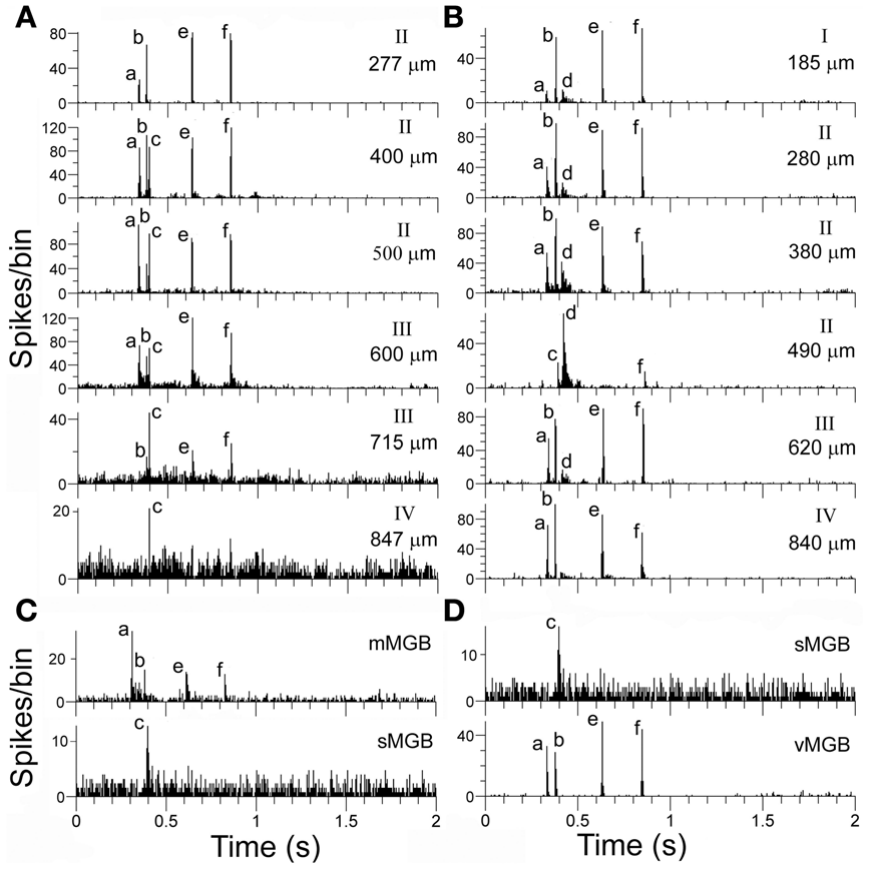
**(A, B)** Responses from a series of units from two separate orthogonal tracks in AI(LF). The depth of each unit and the probable cortical layer are indicated at the right of each panel. **(C)** The top histogram shows the response of a unit in the medial MGB that corresponds to the response of the first unit in the cortical track **(A)** while the bottom panel shows the response of a unit in the shell MGB that corresponds to the response of a unit that may be in layer IV. The cortical units recorded in the middle of track **(A)** appear to show a summating convergence of the responses recorded at the top and bottom of the track although the response at 715 μm also appears to have an initial strong suppression of any response. Responses of track **(B)** units in layer IV (840 μm) and layer II (490 μm) correspond to responses found in the MGB and shown in **(D)**. Other units in the track again seem to show a summative convergence.

The 11 tracks with dissimilar responses can be most parsimoniously explained by the suggestion that there are two distinct thalamic inputs, which could either remain separate or be combined with each other, to give a response that had elements of both inputs. Examples of this are given in the two tracks shown in Figure 10, which also shows examples of corresponding units in the MGB. In the first unit recorded in the track shown in 10A (277 μm) there is a clear response to elements (a, b, e, and f). This is a common cortical response and was recorded in the cortex of 8 separate animals as well as in the MGB. An example from the medial MGB is shown in the first panel of Figure 10C. By contrast, a unit thought to be in layer IV of the track in 10A (847 μm) only shows a small response to element (c). A corresponding response was recorded in the shell division of MGB as shown in the last panel of Figure 10C. At intervening depths along the cortical track there was a combination of the individual responses recorded from the start and end of the track so that units responded to elements (a, b, c, e, and f) (apart from the unit at 715 μm where the response to the (a) element was suppressed). The track shown in Figure 10B is from a different animal, but can also be explained by a possible combination of responses produced by different thalamic inputs. The unit at 840 μm (probably layer IV) has a strong response to elements (a), (b), (e), and (f) whereas the unit at 490 μm has a strong response to element (d) and a small response to (c) and (f). The other units in this track all have a combination of these two responses although the response to element (c) was suppressed. The input to layer IV would be expected to come from the ventral MGB and an example of a vMGB unit with the same response pattern as the layer IV unit is shown in the last panel of Figure 10D, along with the unit in the MGB shell that only responded to element c. There was no indication that the responses which seemed to be a result of a combination of the inputs driving different nearby cells were associated with any particular layer.

**Figure 11:**
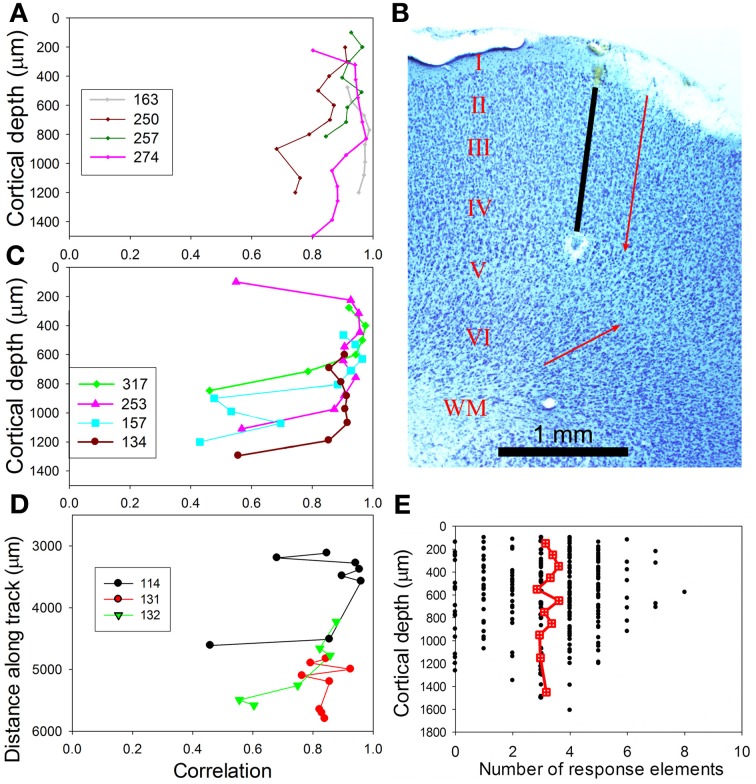
**(A)** Similarity of recorded responses in tracks across AI(LF). For any one track the mean PSTH was correlated with the individual responses and the correlation values plotted against the distance along a track. These four tracks were oriented in a direction orthogonal to the cortical surface and were representative of eight tracks that mainly showed similar values over a distance of about 1 mm. **(B)** Coronal section through AI(LF), stained for Nissl substance and showing the position of an orthogonal electrode track (black line) that has been extrapolated between the blood filled start of the track and an electrolytic lesion at the upper edge of layer V. The parallel red arrow indicates the orientation of the stacks of cells separated by bundles of myelinated fibers that indicate the orientation of cortical microcolumns in the outer layers. The red arrow pointing obliquely upwards indicates the orientation of cell columns in the deep layers. The cortical layers are indicated by Roman numerals and the white matter by WM. Part of layer I, above the red arrow, was injured during the experiment. **(C)** A further four orthogonal tracks that all show at least one sudden change in the type of response. **(D)** Three tangential tracks that also show a sudden change in response type if the track extends for more than about 1 mm. **(E)** Scattergram plot showing the number of elements in chutter that units respond to over the range of depths sampled. The red square/cross symbols joined by a line indicate the running mean values for the number of elements and these remain fairly constant over the depth of AI(LF) sampled.

The degree of similarity within a track was measured by calculating the correlation between the PSTH of a given unit and the mean PSTH for the track. Examples of four orthogonal tracks with similar responses in layers II–IV are shown in Figure 11A while tracks with similar response types in layers II and III, but a sudden change in response type within layers I, IV, or V, are shown in Figure 11C. The PSTHs for one of the tracks (250) in Figure 11A were shown in Figure 9A. When the distribution of correlation values for the uniform group was compared to the group with sudden changes there was a large difference in the standard deviation and in the mean values. Thus, the mean correlation value for the uniform group was 0.89 (s.d. 0.07) while the mean correlation value for the sudden change group was 0.77 (s.d. 0.20) and the probability of the two populations being the same was very small (*P* = 0.00001). Three tangential tracks were also made and these also tend to show similar responses for a distance of up to 1 mm before there is a sudden change in response type (Figure 11D). We were unable to record from orthogonal tracks which traversed all six cortical layers. When lesions were made to check the orientation of our orthogonal tracks these were found to be reasonably well-aligned with the strings of cells which are separated by bundles of myelinated fibers (compare the black electrode track and the red arrow in Figure 11B). However, the strings of cells in layer VI had a very different orientation (compare lower red arrow). This meant that electrodes that went into layer VI were very likely to be in a different column from that in which the units in the upper layers were recorded. This may explain some of the sudden changes in type of response in tracks that were deeper than about 1000 μm. The presence of functional columns or modules with a diameter of about 800 μm that are mainly centered on layers I–IV is consistent with our previous study of AI(LF) which concluded that there are functional columns of this size (Wallace and Palmer, 2009) and is the same size as the hypercolumns of the primary visual cortex (Hubel and Wiesel, 1977).

The number of elements, to which a unit responded, was plotted against cortical depth as shown in Figure 11E. There was no significant change in the number of elements to which units responded over the range of depths measured (layers I–V). This is consistent with the hypothesis that some response combinations are present in a cluster of cells that stretches across two or three cortical layers and where all the cells have the same thalamic inputs. The alternative hypothesis—that cells with simple responses to a few of the elements are located in layers III/IV and more complex responses involving constructive convergence are present in layers II or upper V was not supported. There was no evidence of the simpler combination types (one or two elements) being associated with a particular cortical layer. Most simple types were recorded at a range of depths corresponding to layers I through IV or V.

**Figure 12:**
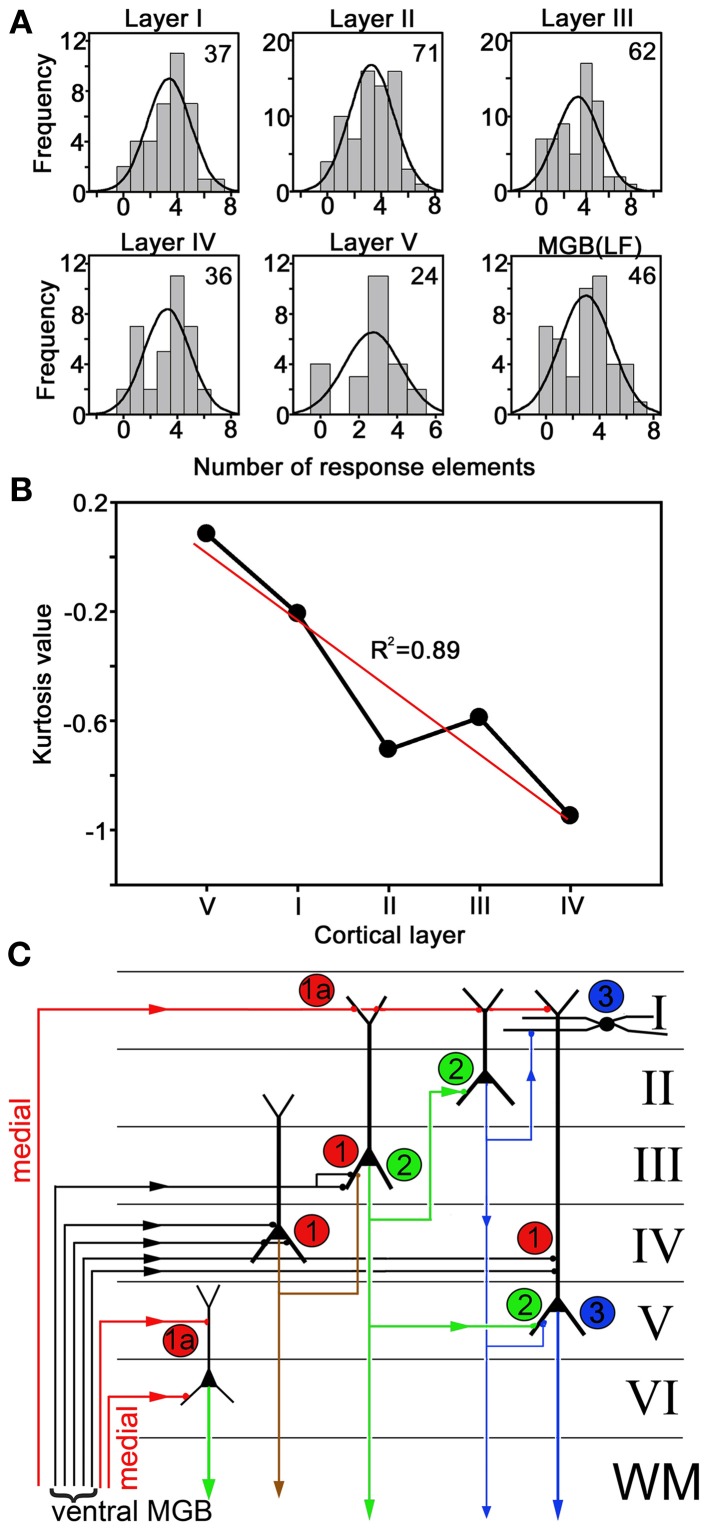
**(A)** Units were assigned to a cortical layer based on their depth and the number of units responding to a given number of call elements was plotted as a histogram and compared to a normal distribution. The relatively small number of units recorded from each layer (number in upper right hand corner of each panel) meant that the distributions were not perfectly normal, but it was possible to calculate a value for how flat the distributions were (kurtosis). **(B)** Plot of kurtosis values for the distributions of response frequency to a particular number of call elements versus the cortical layer. When the layers were arranged in functional distance from layer IV there was a linear relationship. The regression line (red) had an R^2^ value of 0.89. **(C)** Simplified diagram showing some of the main excitatory connections in the auditory cortex. The main thalamic inputs from the ventral MGB are thought to terminate on a variety of cells in layer IV and the base of layer III (labeled 1) whereas the inputs from the medial MGB (labeled 1A) mainly terminate in layers I, V, and VI. Layer IV pyramidal cells project into layer III whereas layer III pyramidal cells project to layers II and V (labeled 2). Layer II pyramidal cells project to layer I and layer V (labeled 3).

To analyse the laminar changes in response type in more detail we assigned all units to a cortical layer based on our previous measurements of AI(LF) laminar depth (Wallace and Palmer, 2009). This allowed us to plot the frequency distribution for the number of units that responded to a particular number of elements in each layer. The number of units in each layer was not very large, but it was assumed that they were normally distributed and a normal curve was superimposed on each distribution (Figure 12A). When a One-Way ANOVA was performed on the mean number of elements responded to in each layer there was no significant difference (*F* = 0.626; *P* = 0.645). Some of these curves were flatter or more peaked than others and when a measure of this (kurtosis) was plotted against lamina there were clear differences (Figure 12B). The kurtosis value for MGB(LF) (−0.83) was similar to that for layer IV (−0.95) and the kurtosis value became more positive (more peaked) in layers progressively more distant from layer IV. The exception to this is layer V which had the most positive kurtosis value. However, there are not thought to be any significant excitatory connections between layer IV and V of the cortex and although they are adjacent the excitatory pathways between them are thought to be di- or trisynaptic (Wallace and He, 2011; Wu et al., 2011). This is illustrated in Figure 12C which shows a simplified circuit diagram for excitatory connections in the AI. When the kurtosis values are plotted starting with layer V (as the layer synaptically furthest from the thalamorecipient layer IV) and then layers I–IV there is a linear relationship with a correlation coefficient of 0.89. This indicates that there are laminar differences in the responses to the chutter with layer IV being closest to the ventral thalamic input and layers I and V being the most different.

## Discussion

### Thalamo-cortical transformation in AI

This study investigated the neural representation of a call sequence comprised of acoustically different syllables in two, frequency-matched, brain areas AI(LF) and MGB(LF). We have previously suggested that the low-frequency end of AI (up to 1.5 kHz) is a separate zone of AI because it contains within it many units that are sensitive to interaural time differences and some of which show phase-locked responses to pure tones. The area of AI containing units with CFs of >1.5 kHz does not respond to interaural time differences or show phase-locked responses (Wallace and Palmer, 2009). AI(LF) appears to be particularly sensitive to low-frequency calls such as purr and chutter and gives strong isomorphic responses to purr (Wallace et al., 2005). It has previously been shown that cortical neurons with CFs of up to about 20 kHz can respond to chutter when it is presented at high sound levels (Wallace and Palmer, 2008; Suta et al., 2013). This is apparently because elements such as (c) and (d) contain sharp, noisy transitions which contain significant energy at up to 22 kHz. These high-frequency neurons have different responses from the low-frequency neurons.

By investigating the representations of neural responses between the MGB and AI for the chutter sequence, we hoped to determine what sort of computations were being made in AI. However, thalamic and cortical responses were very similar both on an individual and group basis. Some cells in both structures could respond selectively to a single element within the call sequence and units in both structures rarely responded to all elements unless they were presented at a high sound level. Although some cortical units (~10%) responded to a single element, the majority showed a wide range of different cortical response combinations to the 8 main elements in the chutter. This indicated that there had been a large amount of filtering of the response somewhere between the auditory nerve and the cortex, but it did not seem to occur at the thalamocortical level. The responses at the level of the cochlear nerve are generally very faithful representations of stimuli and include all the temporal elements that contain energy within the range of their narrow spectral filter (Palmer et al., 1995). All of the 8 elements of the chutter contained low-frequency energy that should have been sufficient to stimulate the auditory nerve fibers that were the origin of the input for the AI(LF) cells in this study. On the basis of previous studies of the chutter call in the guinea pig inferior colliculus, thalamus and AI (Suta et al., 2003, 2007, 2013) it appears that most of the alteration in the chutter representation occurs at the level between the inferior colliculus and MGB. Many cells in the inferior colliculus provide accurate representations of the waveform of calls such as chutter, but up to 30% of these may be GABAergic and provide an inhibitory input to the thalamus (Winer et al., 1996). Interactions between these inhibitory and excitatory inputs may provide a range of responses at the level of the thalamus where fewer call elements produce a response than in the midbrain. The AI may also be involved in producing the variety of responses to chutter because of its profuse corticofugal output (Winer, 2005). Recordings in the guinea pig thalamus have shown that many thalamic cells receive a modulatory input from the cortex (He et al., 2002) and that this can modulate the temporal firing pattern. For most neurons in the MGB the cortical input is excitatory, but inhibitory inputs also occur, particularly in the extralemniscal divisions (Yu et al., 2004). Out of the 244 units in AI(LF) that gave a discrete response to chutter, 214 units (88%) responded to element (a), the first reasonably loud element of the call. The next element (b) did not start until about 20 ms later and this is ample time for corticofugal modulation to become active and to modulate responses to any of the temporal elements from (b) onwards. To find out if the corticofugal pathways are important in generating the wide diversity of response combinations to chutter, it will be necessary to record from units in the thalamus before and after inactivating the cortex (Villa et al., 1999; Palmer et al., 2007). We have not yet done this.

Previous work on the chutter representation in the MGB (Tanaka and Taniguchi, 1991; Philibert et al., 2005; Suta et al., 2007) had led us to expect a limited variety of combination responses in the thalamus. These could then be processed by intrinsic circuits in the cortex, involving inhibition of selected elements (Gaucher et al., 2013a), or convergence of different thalamic inputs in order to produce a much greater range of cortical response combinations. Some of our orthogonal tracks were consistent with the idea of convergence from different thalamic inputs and inhibition is undoubtedly involved, but overall the variety of chutter response combinations identified in the thalamus appeared to be just as great as in the cortex. Previous work on the guinea pig auditory system had also failed to find any striking differences between the thalamus and AI when either waveform envelope representation or spike timing reliability were compared (Huetz et al., 2009). The only significant difference we could detect was in the distribution of response elements in the different layers of AI. The distribution in MGB and layer IV of AI appeared very similar whereas the distribution for layers I and V had a more positive kurtosis indicating a higher proportion of responses to three or four elements. This may be evidence of intrinsic processing involving both local inhibition and convergence which when added together don't produce any significant change in the mean number of elements responded to, but does reduce the number of responses to a high or low number of elements. Optical imaging studies of the anaesthetized guinea pig AI have shown that intrinsic connections are still active and allow the propagation of activity within an isofrequency column following auditory or direct electrical stimulation (Song et al., 2006).

In the visual cortex thalamic afferents terminate on granule cells in layer IV and by constructive convergence produce “simple” bar detectors that are unlike anything found in the thalamus. These granule cells then project to the superficial and deep layers and by processes such as convergence apparently produce responses in these other layers that are more “complex” and respond to features such as a corner (Hubel and Wiesel, 1977; Martinez et al., 2005). By contrast, there appear to be comparatively few granule cells present in layer IV of the AI in the cat (Smith and Populin, 2001) or other species and the circuit of intrinsic cortical connections has still not been properly elucidated (Wallace and He, 2011; Wu et al., 2011). Thalamic afferents are thought to mainly terminate on layer III pyramidal cells (Douglas and Martin, 2004), but may not be specific for any particular cell type. Thus, most of the cells we recorded from in layers II–IV may have had a direct thalamic input and this would help to explain why there was often so little variation in the responses to chutter as an electrode passed in an orthogonal direction through the upper cortical layers. There are probably also vertically arranged axon collaterals, coming from the pyramidal cells in the upper layers, which help to bind together the activity within a cylinder of cortical cells (Wallace and He, 2011). Recent work has shown that clonally related neurons form radial strings, in the mouse visual cortex that have the same orientation selectivity (Li et al., 2012) and radial strings of functionally related cells may also occur in the AI.

### Distributed responses to chutter within multiple cortical modules

A previous study of guinea pig cortex (Creutzfeldt et al., 1980) and more recent studies in the marmoset (Wang et al., 1995) and cat (Gehr et al., 2000; Eggermont, 2001) have suggested that individual calls are represented by distributed networks of neurons that are temporally synchronized. These network theories of call representation are related to the group selective theory of brain function (Edelman and Mountcastle, 1978; Tononi et al., 1999). Edelman suggested that structures such as the AI are composed of large repertoires of modules each of which has slightly different response characteristics. Many of the modules may respond to a given call, but they would do so in varied and distinctive ways. This theory is based on the expectation that there is a relatively small degree of redundancy within cortical responses and so finding large numbers of identical responses to a call sequence would be inconsistent with this view. Recent work has shown that at the level of individual neurons there is relatively little redundancy in the response to conspecific calls such as chutter partly as a result of local inhibition within AI (Gaucher et al., 2013a). Our results were consistent with other work showing that the temporal sparse code found in AI is a first step in generating a high level representation of conspecific vocalizations (Gaucher et al., 2013b). Although individual cells in AI may not give a complete representation of an irregular call like the chutter they are thought to be able to represent an object through temporal coherence (Elhilali et al., 2009). Even some of the more unusual units that only respond to some of the chutter components will contribute to the population representation of the whole call, since their responses are precisely timed (Lakatos et al., 2005).

We have shown that a call sequence such as the chutter evokes responses from many cortical cells, but there is a large diversity in the combination of elements to which each cell responds. This diversity seems to be a reflection of the way that the mammalian brain has evolved, in order to process a broad range of acoustic inputs, rather than evidence that the guinea pig is attaching any particular significance to the temporal order (syntax) of the elements. In some species, such as bats (Esser et al., 1997; Gadziola et al., 2012), the syntax of sound elements does appear to be important, but in the guinea pig there is little evidence that individual variations in a call has any behavioral significance (Berryman, 1976, 1981). The chutter seems to represent a form of babble rather than a meaningful sequence that can be consciously modulated.

The relative lack of variation across the upper layers may partly be a result of the anesthetic because the presence of anesthetics can radically alter the responses to vocalizations such as chutter in the guinea pig AI (Syka et al., 2005) and there is greater spike-timing reliability in the cortex of awake animals (Huetz et al., 2009). The processing occurring in awake animals may be more complicated and subject to processes such as the sleep/wakefulness cycle (Edeline et al., 2001; Issa and Wang, 2008) or attentional modulation (Fritz et al., 2005). Despite the limitations of this study we feel that it still provides support for the hypothesis that units in AI have a propensity for splitting the continuous acoustic input into individual acoustic objects. This is in contrast to the small rostral belt area (area S) where units make a much more complete representation of the chutter sequence (Grimsley et al., 2012).

### Conflict of interest statement

The authors declare that the research was conducted in the absence of any commercial or financial relationships that could be construed as a potential conflict of interest.

## Abbreviations

AI(LF): low-frequency part of primary auditory cortex
CF: characteristic frequency
MGB(LF): low-frequency part of medial geniculate body
PSTH: peristimulus time histogram
SPL: sound pressure level.
